# High Resolution Micro-Computed Tomography Reveals a Network of Collagen Channels in the Body Region of the Knee Meniscus

**DOI:** 10.1007/s10439-021-02763-6

**Published:** 2021-04-07

**Authors:** Greta Agustoni, Jared Maritz, James Kennedy, Francesco P. Bonomo, Stéphane P. A. Bordas, Olga Barrera

**Affiliations:** 1 Department of Health Science and Technologies, ETH, Zurich, Switzerland; 2 University of Oxford, Oxford, UK; 3 Università degli Studi di Palermo, Palermo, Italy; 4 Institute of Computational Engineering Sciences, University of Luxembourg, Luxembourg, Luxembourg; 5 Institute of Research and Development, Duy Tan University, K7/25 Quang Trung, Danang, Vietnam; 6 Cardiff University, School of Engineering, Cardiff, UK; 7 School of Engineering, Computing and Mathematics, Oxford Brookes University, Wheatley Campus, Oxford, OX33 1HX UK; 8 Department of Medical Research, China Medical University Hospital, China Medical University, Taichung, Taiwan

**Keywords:** Keywords: Meniscus, Microstructure, Porosity, Collagen, Micro-CT

## Abstract

The meniscus is an integral part of the human knee, preventing joint degradation by distributing load from the femoral condyles to the tibial plateau. Recent qualitative studies suggested that the meniscus is constituted by an intricate net of collagen channels inside which the fluid flows during loading. The aim of this study is to describe in detail the structure in which this fluid flows by quantifying the orientation and morphology of the collagen channels of the meniscal tissue. A 7 mm cylindrical sample, extracted vertically from the central part of a lateral porcine meniscus was freeze-dried and scanned using the highest-to-date resolution Microscopic Computed Tomography. The orientation of the collagen channels, their size and distribution was calculated. Comparisons with confocal multi-photon microscopy imaging performed on portions of fresh tissue have shown that the freeze-dried procedure adopted here ensures that the native architecture of the tissue is maintained. Sections of the probe at different heights were examined to determine differences in composition and structure along the sample from the superficial to the internal layers. Results reveal a different arrangement of the collagen channels in the superficial layers with respect to the internal layers with the internal layers showing a more ordered structure of the channels oriented at 30$^{\circ }$ with respect to the vertical, a porosity of 66.28% and the mean size of the channels of 22.14 $\mu {\text {m}}$.

## Introduction

The meniscus is a type of tough fibro-cartilage soft tissue that conforms to the surfaces of the tibia and the femur. There are two menisci: lateral and medial meniscus, each one rest between the thigh bone (femur) and shin bone (tibia).^2^ They play a critical role in load transmission to the articular cartilage, structural stability and shock absorption in the knee joint by helping to protect the articulation from the stresses originated during physical activities.^21^

Due to an aging and more active population, the number of meniscus-related operations has markedly increased in the last decade.^20^ The meniscus has variable levels of perfusion. The outer third is well perfused and heals well when damaged, however the inner third, which is not vascularised, does not heal. Repair strategies, like sutures, staples and anchors, allow preservation of the meniscal tissue, however healing of the tissue is more likely to occur when the tear is located in the vascularised area.^26^ On the other hand, degenerative tears tend to start in the inside circumference of the meniscus which is avascular. In this case tears are usually treated by partial meniscectomy. It has been noticed that the loss of the meniscus leads to joint degeneration and osteoarthritis as contact stresses on the tibial plateau increase proportionally with the amount of meniscus tissue removed. Hence replacing the resected meniscal tissue by an artificial implant is necessary in order to avoid the articular cartilage degeneration.^18^ Unfortunately, the clinical and functional outcomes for these devices are not ideal. It is thought that this is primarily due to their inability to replicate the function of the native meniscus. Therefore, mimicking the native biomechanical and structure characteristics of the menisci seems to be the key factor in meniscus replacement functioning.

The meniscus is mainly composed by a fluid phase (60–70% is water) and a porous solid phase which consists predominantly of collagen type 1 for 15–25%.^2^ Petersen and Tillmann^24^ observed a different arrangement of collagen fibers in the cross-section of the meniscus. The first one, the superficial layer is constitute by randomly oriented thin fibrils with about 30 nm diameter. The second layer beneath the surface is made of lamella-like collagen fibril bundles and the more internal layers exhibit collagen fibers running along the circumferential direction which are believed to be responsible of the load bearing capacity of the tissue. A more detailed study on the internal layers of the meniscus has shown additional details on the collagen fibers in the radial directions in the internal regions of the tissue. Radial tie collagen fibers have been discovered to divide the cross section of the meniscus into honeycomb-like compartments which have been observed to contain collagen fibrils (diameter of 5 $\mu {\text {m}}$).^27^ However these results are limited by the imaging techniques used and the related sample preparation which inevitably introduce artefacts such as fixation, mechanical and chemical peeling and dehydration.

Recently a number of advanced imaging techniques have been used in order to characterize meniscal properties as diffusivity,^14^,^31^ biochemical composition^1^ and structural deterioration of tissues.^15^

The three dimensional structure of the “honeycomb-like” compartments have been recently observed in fresh and untreated human meniscal samples.^32^ The authors detected “honeycomb-like” compartment of size varying from micro (25 to 100 $\mu {\text {m}}$) to macro (600 $\mu {\text {m}}$ to 1 mm). Each honeycomb compartment is discovered to be filled by pores. The 3D reconstruction of the pores show essentially collagen channels.

**Figure 1:**
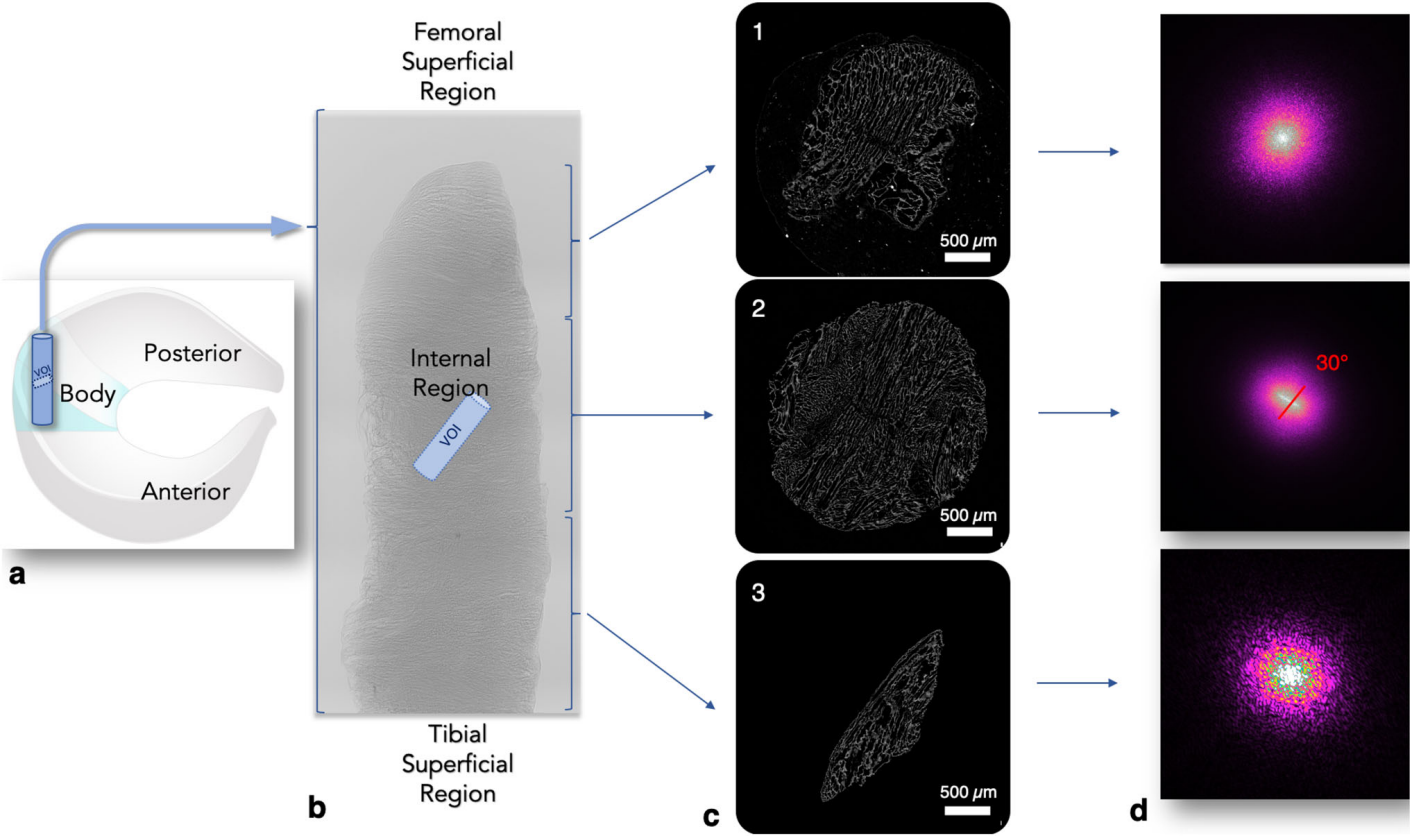
(**a**) Schematic representation of the meniscus, the extracted and scanned cylindrical sample. (**b**) 3D reconstruction of the cylinder and the orientation and position of the VOI extracted within the internal region; (**c**) examples of micro-CT scan images of the architecture of the three regions: (1) femoral superficial region, (2) the internal region and (3) the tibial superficial region; and (**d**) FFT spectrums of (1)–(3) showing that the collagen channels have no specific orientation in the superficial regions and they oriented at 30$^{\circ }$ in the internal region.

The aim of this paper is twofold. Firstly, to confirm by to-date highest resolution Micro Computed Tomography the collagen architecture of the different layers of the body of the meniscus. Secondly, to quantitatively describe for the first time the three dimensional meniscal porous architecture including the statistical distributions of dimensions, frequency and tortuosity of the collagen channels. An overview of the study is shown in Fig. 1. A cylindrical sample from the body of a porcine meniscus was extracted and scanned. Subsequently a Volume Of Interest (VOI, Fig. 1b) in the direction of the orientation of the collagen channels (30$^{\circ }$) has been studied in order to quantify the porous microstructure of the internal layers.

## Materials and Methods

### Sample Preparation

One sample of 33.7 mg (Fig. 1a) was extracted from the central area (body), along the vertical axis of a lateral porcine meniscus using a biopsy punch. Afterwards the sample was immediately frozen at $-\,80\,^{\circ }$C for 12 h and subsequently freeze-dried by setting the parameters shown in the Table 1 in a Benchtop Freeze Dryer (FreeZone Triad Cascade, Labconco) at a vacuum pressure of 0.120 mbar. The resulting weight and dimensions were 10.2 mg, 2 mm diameter and 7 mm height.

**Table 1:** Freeze-dry process settings.

Time (h)	Shelf’s temperature ($^{\circ }$C)
0	$-35$
1	$-10$
1.5	0
1	10
1	20
1	37
0.5	STOP

### Micro-Computed Tomography

**Figure 2:**
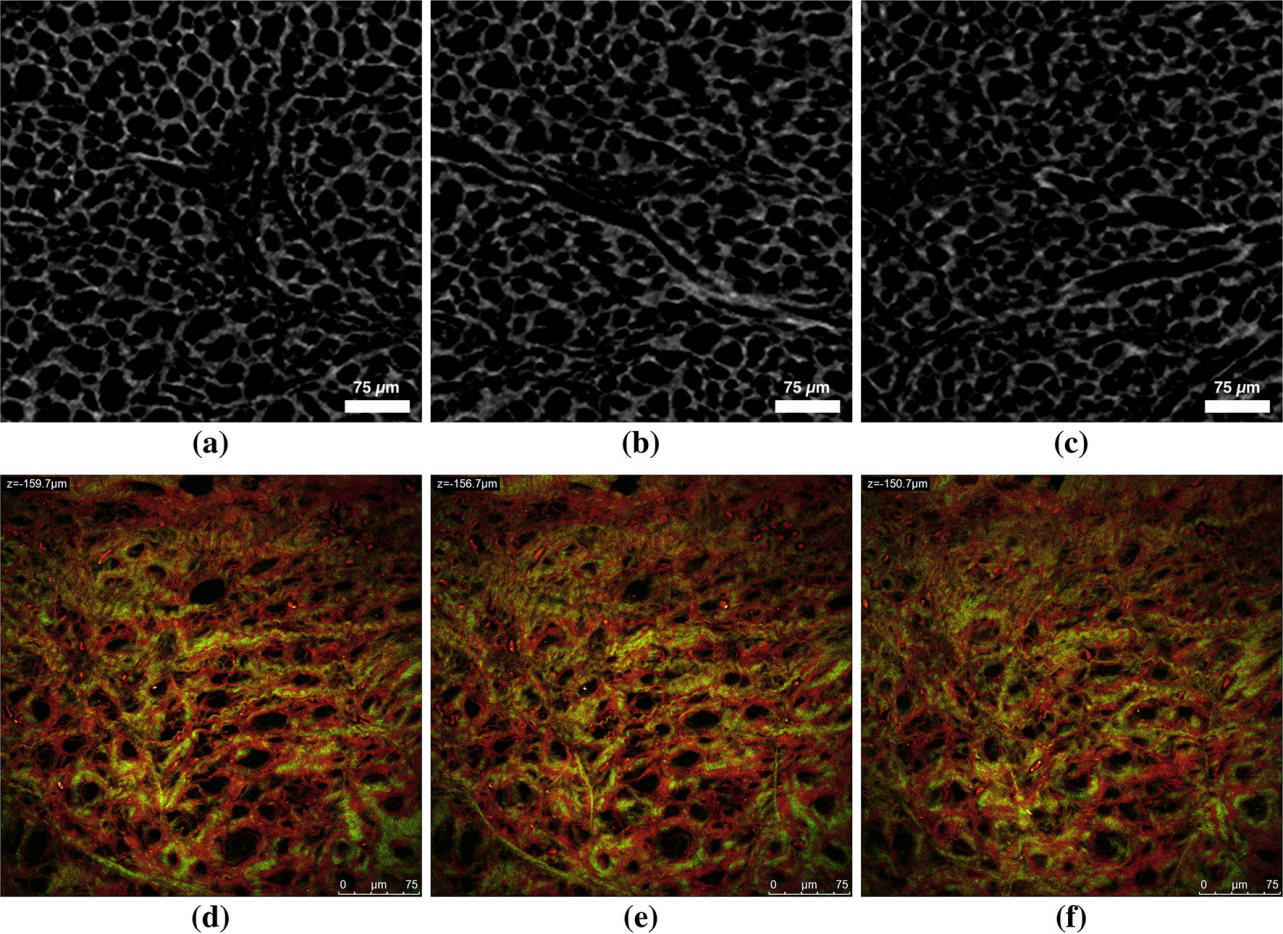
(**a**)–(**c**) Micro-CT scans extracted from the VOI (Fig. 1b); and (**d**)–(**f**) multiphoton Microscopy Images of a similar region of the VOI. In order to carry out multiphoton microscopy, samples were imaged at $1024\times 1024$ pixel resolution using a Leica TCS SP5 laser scanning microscope with a $\times 40$ oil objective (Leica Microsystems, Germany) with a scanning frequency of 400 Hz. Images stack were acquired with steps ranging from 0.5 to 1.7 $\mu$m, along the *z* axis. The two-photon excitation (Spectra-Physics Mai-Tai Ti:Sa ultra-fast laser) was set at 880 nm. The SHG signal was detected in the range of 390–460 nm (green channel) and tissue autofluorescence was detected in the range of 485–650 nm (red channel).

A micro-Computed Tomography ($\mu$CT) scanner (Skyscan 1272, Bruker Kontich, Belgium) has been used to run the $\mu$CT analysis. One base (the tibial side) of the sample was fixed at the base of the instrument rotating support by laying a layer of wax. The images were acquired by setting a rotation step of 0.3$^{\circ }$ with a pixel size of 600 nm. An X-ray beam with an energy level of 40 and an intensity of 250 mA was used. In order to image the whole sample in high resolution mode the functions ’oversize’ and ’batch Scanning’ were used. These functions allow to scan the whole object by dividing it into sub-objects and then reconstruct the sample. In this case the micro CT of the full cylinder has been achieved by combining 5 scans of sub-cylinders. The obtained dataset after the acquisition step consisted of images shown in Figs. 2a–2c in 16-bit tiff format ($1640 \times 2452$ pixels). In Figs. 2a–2c a porous structure is visible, each of these pores present on the image represent a channel in 3D. It is interesting to note that multi-photon microscopy investigations on fresh and non treated samples^32^ showed architectural quantitative results in terms of mean porosity comparable to the freeze dried tissue as reported in Figs. 2d–2f.

The micro-CT scans of the vertical cylinder shown in Fig. 1b account to 11,000 2D images. 3D reconstructions and subvolume extraction have been performed with 3D.SUITE software part of the Skyscan system. 3D.SUITE software has been used to extract a VOI (i.e. a smaller cylinder) in the radial direction from the middle region of the vertical cylinder. The VOI dataset consists of 1002 2D images.

### Fast Fourier Transform Analysis of the Collagen Channels

The Fast Fourier Transform (FFT) is a useful image processing tool to analyse characteristic feature of the spatial domain of an image.^25^ A 2D image is converted from its spatial domain into the Fourier Domain and decomposed into its sine and cosine components. Each point in the Fourier Domain represents a particular frequency which is contained in the spatial domain of the 2D image. With the conversion into the Fourier Domain, it is possible to find the dominant frequencies of the image which influence its geometric structure in its spatial domain.^11^ Gwyddion software (open source) has been used to carry out 2D FFT Analysis of micro CT images and to obtain the related Fourier spectrum. The procedure is described in Reference 32.

## Results

### Collagen Channels Orientation Analysis

Fast Fourier Transform (FFT) Analysis of 11,000 micro-CT images of the whole cylinder in Fig. 1b has been carried out. The collagen fibers are seen to be hollow cylinders i.e. channels. These channels appear to follow different paths in the superficial and internal layers. Figure 1c shows an example of images of the three different regions. The quantification of collagen fibers orientation is perpendicular to the direction of where FFT maxima aligns (FFT spectrums of the internal region in Fig. 1d).^11^ The superficial region from the tibia, shows no channels alignment for the first 0.84 mm of the sample. The channels seem to follow random orientations. The second region (the internal region) shows a consistent alignment of the collagen fibers with an orientation of 30$^{\circ }$ respect to the vertical (see FFT spectrum in the Fig. 1). The region facing the femur, shows as the first region next to the tibia no fibers alignment but for a lesser extent, it covers only 0.04 mm of the probe. A perpendicular cut (radial direction) of the internal region is represented in Fig. 1b (VOI). The architecture of the VOI has been imaged with two advanced imaging techniques (micro-CT and multiphoton microscopy) as shown in Fig. 2. The quantification of a number of parameters of this porous region (VOI) is discussed in detail in the next section.

### Porosity Analysis of the VOI

**Figure 3:**
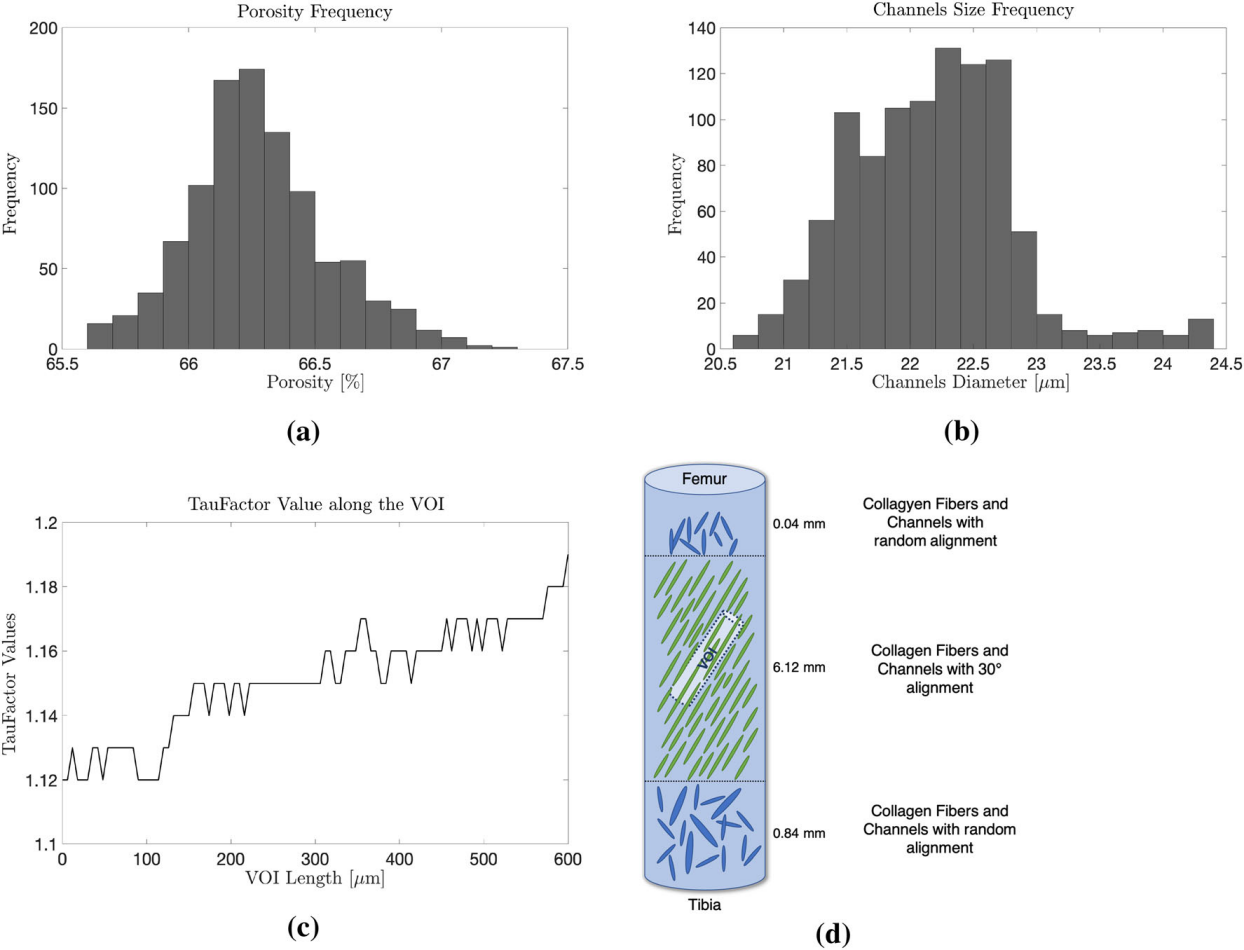
Results of the porosity analysis of the VOI. (**a**) Porosity Frequency of the VOI (Frequency defined as the amount of micro-CT images of the VOI); (**b**) Channels Size Frequency along the VOI (Frequency defined as the amount of channels having the same diameter); (**c**) Results of the Tortuosity Analysis using “TauFactor”—TF Values along the VOI length; and (**d**) structure of the vertical cylincer and collagen fibers orientation.

1002 Micro-CT images obtained from the VOI were processed and analysed using a MATLAB Code (see “Appendix”). With the MATLAB script it was possible to calculate different parameters such as porosity and its variation along the VOI, pores’ diameter and its frequency throughout the VOI. Firstly we measured the mean porosity of the VOI which accounts to 66.28% (standard deviation of 0.27). Figure 3a shows the porosity frequency defined as the amount of micro-CT images of the VOI showing the same values-range of porosity, ranging from 65.6% (lowest value measured) to 67.2% (highest value measured). Secondly we measured the pore size and the frequency of the pores having the same diameter. 22.14 $\mu$m represents the mean diameter measured (standard deviation of 13.05). Figure 3b displays the amount of pores having the same diameter which have been identified along the VOI. It shows that the majority of the pores has a diameter between 21.96 and 22.76 $\mu$m.

### Tortuosity Analysis of the VOI

A tortuosity analysis is performed to understand the path of the channels seen in the 3D reconstruction of the VOI. The MATLAB Application “TauFactor” enabled us to carry out the tortuosity analysis. An average value of TF = 1.15 has been measured along the VOI. Figure 3d shows the values of TF along its whole length. In the first micrometer of the VOI a lower TF value has been calculated, which indicate a less convoluted geometry of the channels. In the rest of the VOI greater values of TF are calculated, representing a more tortuous flow path than in its first 300 $\mu$m.

## Discussion

By using the Gwyddion software was possible to execute FFT transforms of the micro CT images obtaining their respective FFT spectrums. The latter confirmed our expectations because the channels’ orientation is also directly visible from the micro-CT images (Fig. 1c). Fast Fourier Transform has been already used in various studies to analyse and describe collagen fibers’ orientation or just fibers’ alignment of many materials, the majority of the works are based on skin samples. In the work of Wu *et al*.^33^ a quantitative analysis on collagen morphology of aged skin has been carried out. By using FFT they were able to understand the texture of skin based on its collagen orientation’ index and bundle packing. De Campos Vidal and Mello^6^ focused on porcine chordae tendinae with the aim to understand the orientation and undulations of collagen fibers. They carried out a FFT Analysis of the collagen structure, technique that enabled them to identify an helical structure present in the chordae tendinae. Furthermore, Fisher *et al*.^9^ developed a novel electrospinning method to produce meniscal nanofibrous scaffolds. A FFT analysis enabled them to analyse the spatial changes in fiber alignment present in the scaffold. These studies confirmed that the use of FFT in determining structure pattern orientation is a very reliable and well-known technique to quantify this characteristic. There are no studies that used high-resolution micro-CT scans as imaging technique combined with a FFT analysis to quantify collagen or soft tissue pattern orientation. The majority of the works that applied FFT to the analysis of structure orientation have mostly used multiphoton microscopy.^32^,^33^ One of the first studies on the macroscopic structure of the meniscus is the one of Bullough *et al*. Its results describe that along the circumferential direction, collagen fibers are arranged to resist the tension and the fibers along the radial direction act as tie fibers in order to avoid a longitudinal splitting of the meniscal tissue.^4^ Petersen *et al*. brought some novelties regarding the arrangement of the radial collagen fibers.^24^ They describe 10 $\mu$m bundles arranged collagen fibrils in the radial direction which associate laterally to form a honeycomb network within the body region of the meniscus.^24^ Although the important findings of these studies, the techniques of preparation of the samples used such as fixation, dehydration and chemical peeling can alter the tissue structure limiting the quantification of the results.^10^ Therefore, the use of non-invasive micro-CT systems for the analysis of the sample is a proved method for the quantification of tissues properties. The circumferential arrangement of the collagen fibers presented by Bullough *et al*.^4^ and Petersen and Tillmann^24^ has been confirmed by Vetri *et al*.^32^ Otherwise regarding the radial section, Vetri *et al*. brought novel results showing that collagen bundles form the side walls of highly porous honeycomb-like structures developing in space as tiny channels.^32^ Although multiple studies attempted to elucidate the structure of the meniscus in both circumferential and radial directions, there is no evidence of the meniscal composition, in particular of the collagen fibers arrangement, in the vertical direction apart this paper. The results presented in this work show a preferred orientation of the collagen fibers in the internal region of the vertical sample. Upon FFT analysis we can divide the sample into three sections having different collagen orientation. Our results complete the description of the collagen pattern in the meniscal tissue, meaning that along the defined directions (circumferential, radial and vertical) the collagen fibers are arranged into a particular design. The channels structure with an orientation of 30$^{\circ }$ is mostly present in the middle of the micro CT image and extends itself for the whole diameter of the sample. At its right and left side, a porous structure similar to the one described and quantified in the next section can be identified.

**Figure 4:**
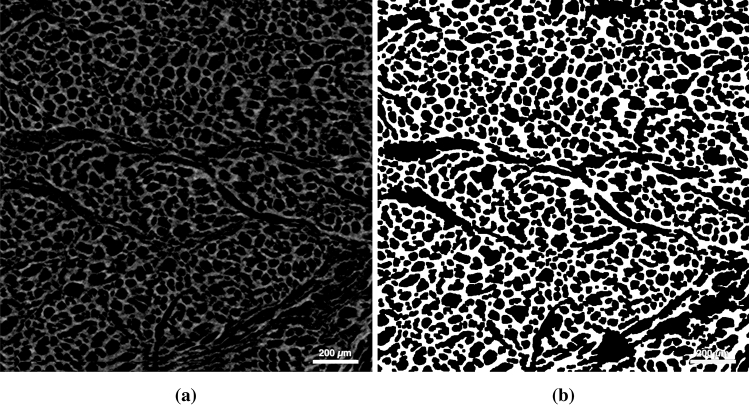
(**a**) Native VOI micro-CT scan, bar = 200 $\mu$m; and (**b**) Processed VOI micro-CT scan, bar = 200 $\mu$m.

High-resolution micro computed tomography uses X-radiations to penetrate any kind of material in order to provide detailed microstructural information.^29^ Technique that belongs to the non-destructive test methods allowing to display internal structure images in a non-destructive manner with a very high resolution. Furthermore, it is also capable of eliminating laborious sample preparation, is less susceptible to artifacts and is cost-effective. Micro-CT systems are optimized to provide very good spatial resolution in order to obtain an image that is very close to the histological microscopy of the material analysed.^13^ The data provided can be presented in two and three dimensions enabling the observation and measurement for different purposes.^29^ Despite its many advantages, micro-CT carries also some limitations, such as the inability to differentiate between structures with a similar atomic density. The radiograph records the absorption of X-rays by a three-dimensional material and project them onto a two-dimensional image with different gray-scale intensities.^29^ If the difference in the absorptive properties of two optically different structures is not sufficient, they will appear to be the same in a micro-CT generated scan.^29^ Furthermore, by using X-radiations results in a potential risks to the research operating staff.^13^ However, the application of micro-CT within material sciences is gaining popularity.^17^ The majority of human-related studies use micro-CT in the quantification of bone density and architecture and in the vascular research. X-ray computed tomography is also a very well-known technique to measure porosity in materials, a lot of works can be found in the domain of concretes, stones and metal implants analysis such as References 7,16,23,28. This paper represents the only work using micro-CT as imaging technique to analyse the microstructure of the meniscus. This imaging method enabled us to obtain high-resolution images in gray-scale intensities in which the channels can be well distinguished from the collagen structure around them, an example is in Fig. 4a.

Micro-CT technique is not ideal to image soft tissue in physiological condition (i.e. wet) as there is no enough contrast between the liquid and solid phase. This is the reason behind the choice of freeze drying the meniscal sample. The MATLAB Script used to execute the porosity analysis has been written upon the characteristics of the micro-CT images at disposition. Depending on color, contrast, size of the objects displayed and parameters to calculate, the code has been adjusted to our scans. There are no Scripts fit for all images, every set of images has to be processed in an optimized way upon its imaging characteristics. The process of finding the suited coding sequences to obtain meaningful results was the main focus for the first part of this study. Although a margin of error is present in this process because during image processing operations a tiny amount of the image information get lost, we still manage to obtain results that are able to explain the real porous structure of the meniscus.

Tortuosity Factor (TF) allows the quantification of the apparent decrease in diffusive transport which results from the convolutions of the flow path through a porous material.^5^,^8^ Tortuosity factor and tortuosity represent two different parameters but they both characterize the geometry of the porous media. Tortuosity ($\tau$) equals the ratio of the actual length of the flow path divided by the length of a straight line in the direction of flow.^12^,^19^ TF and tortuosity both increase proportionally with more tortuous pathways. If the cross-sectional area of the flow stays constant, tortuosity factor is equal to the square of tortuosity.^30^ If the flow path is direct, TF and tortuosity tend to a value of 1 which corresponds to their minimal value. Values greater than 1 represents a more tortuous geometry of the sample which decrease the velocity of the diffusive transport.^3^,^5^ “TauFactor” is a MATLAB application which allows to calculate the decrease in diffusive transport caused by the convoluted geometry of the media. It quantifies the tortuosity factor of interconnected porous phases in a three-dimensional volume along three mutually perpendicular axes.^5^ In our study, the tortuosity analysis has been carried out in the direction of the channels’ flow present in the VOI (radial direction). The micro-CT study of Backeberg *et al*. used the TauFactor Application to quantify the tortuosity of permeable pathways in clay-rich mud stones. They obtained much higher values of tortuosity factor (TF $\le$ 1000) representing a more convoluted geometry of their samples.^3^ Furthermore, the samples having a lower value of tortuosity factor, are the one having a higher percentage of porous phase volume. The values obtained from our tortuosity analysis suggest an almost direct flow path through the VOI in comparison to the values calculated in the study of Backeberg *et al*.^3^ In relation to their results, the high porosity percentage described in our porosity analysis suggest a possible link to the very low convoluted geometry of the VOI. The focus of this work is the body region of the meniscal tissue. The body region is divided into three areas: red (vascular), red–white (partially vascular) and white (avascular). The analyzed sample was extracted from the vascular region which is the area mostly affected by the contact between the femur/tibia cartilage. A complete study of the whole meniscal body architecture would include the analysis of samples extracted from the red–white and white region. As the tissue becomes thinner when approaching the white region a different porosity and collagen channel size/frequency are expected. This is currently part of an ongoing study. The summary of the work and findings is given below:

- A vertical sample from the lateral central meniscal body was taken and analysed to discover a porous structure containing fluid channels.
- These channels are orientated with a 30$^{\circ }$ inclination to the vertical in the internal region, but are randomly aligned close to the tibial and femoral surfaces.
- Channels had a mean size of 22.14 $\mu$m and show an almost straight path in the radial direction.
- The thickness of the tibial superficial layer in which the channels have no specific orientation is greater than the femoral one. Further analysis on the internal region found a porosity of 66.28% in the radial direction.

## Appendix

### Porosity Analysis and Collagen Channels Size Distribution of the VOI

A MATLAB code (see “Appendix”) has been written in order to execute image processing operations for extracting quantitative information of the characteristic features of the microstructure: i.e. porosity along the 3D domain, pore size distribution and their frequency. A detail of the original image of micro CT scan and the processed image to perform the quantitative analysis is shown in Fig. 4.

The first operation we used to processed the micro-CT scans of the VOI is image binarization. Process that enables to convert a gray-scale image into a black-and-white one (binary image) reducing the data contained in the image.^22^ The process is also known as image thresholding which is a form of segmentation in which the image is divided into its constituent objects.^22^ Like others image processing operations, image binarization is not trivial and is dependent on information-content of the image.^22^ The result of the binarization of the micro-CT scans is presented in Fig. 4b. The transformation to binary images was necessary because porosity is defined as the amount of black pixels (pores) over the total amount of pixels in a binary image. By using this definition porosity for every micro-CT scan has been calculated. Subsequently the binary images have been inverted, the pores became white. The white portion of the image is the one containing the major data and with this operation was possible to introduce a structuring element decomposition to recognize the pores. The function “strel (‘disk’, 6)” creates an approximated flat-disk shaped structuring element with a radius of 6 pixels which enables to recognize the presence of circular objects (pores) within the image. By using the function “imopen”, a morphological opening on the binary image with the previously defined structuring element was performed. This operation consist of an erosion followed directly by a dilation, both using the same structuring element. To eliminate the white or black snowflakes present in the image, the function “imfill” has been used. This operation is capable to fill holes defined as a set of background pixels that cannot be reached by filling in the background from the edge of the image. By doing so was possible to obtain homogeneous white pores. At this stage was possible to calculate the radius of every pore by setting a roundness limit of 0.8. Circular structures with a roundness above 0.8 were considered in the process of calculation. 1 Corresponds to a perfect disk.

*Porosity analysis* the images were binarised using the “imbinarize” function. Porosity of each image was calculated by counting the amount of black pixels and dividing it by the total amount of pixels present in the image.

*Pore size and their distribution* the inverted binarised images, pores displayed in white, has been used to calculate the pore size distribution. A disk structuring element with a radius of 6 pixels is defined and used with a morphological filters to remove black snowflakes with a radius smaller than 6 pixels present in the white pores (“imfill” MATLAB function). To define the boundaries of the pores, the function “bwboundaries” has been used to trace the pores’ borders on the inverse binary images. A MATLAB structure with measure properties of each image has been created using the function “regionsprops”. From this structure dimensions such as mean pore diameter and mean standard deviation has been calculated for all the 1002 micro CT images.
